# A new species of *Cinnamomum* (Lauraceae) from the Bladen Nature Reserve, southern Belize

**DOI:** 10.3897/phytokeys.81.13256

**Published:** 2017-06-07

**Authors:** Steven W. Brewer, Gail L. Stott

**Affiliations:** 1 Belize Foundation for Research and Environmental Education, PO Box 129 Punta Gorda, Belize, Central America; 2 Department of Plant Sciences, University of Oxford, South Parks Road, Oxford, OX1 3RB, UK

**Keywords:** Cinnamomum, Lauraceae, Belize, Central America, Bladen Nature Reserve, Maya Mountains

## Abstract

A new species in the Lauraceae, *Cinnamomum bladenense* S.W. Brewer & G.L. Stott, is described from the Bladen Nature Reserve in southern Belize. The new species is similar to *Cinnamomum brenesii* (Standl.) Kosterm., from which it differs by its much smaller, narrowly-campanulate flowers, its inner tepals glabrous abaxially, its shorter petioles, its minutely sericeous younger twigs, and its abaxial leaf surfaces not glaucous and with prominent secondary venation. A description, preliminary conservation assessment, and photographs of the species as well as a key to and notes on the *Cinnamomum* of Belize are provided.

## Introduction

*Cinnamomum* Schaeff. currently contains c. 350 species (Rohwer 1993), primarily in tropical Asia, with approximately 47 species found in the Neotropics (Lorea-Hernández 1996). We follow Lorea-Hernández’ (1996, 1997) use of leaf venation pattern, presence and distribution of domatia along midvein and secondary veins, pubescence type, inflorescence structure, pubescence of floral parts, hypanthium development and persistence of tepals in fruit as some principal features for distinguishing species in the genus. Lorea-Hernández’ revision of the genus for the Neotropics (1996) recognized only one species for Belize: *Cinnamomum triplinerve* (Ruiz & Pav.) Kosterm., a synonym of *Cinnamomum montanum* (Sw.) Bercht. & J. Presl (Rohwer 2014).

**Figure 1.**
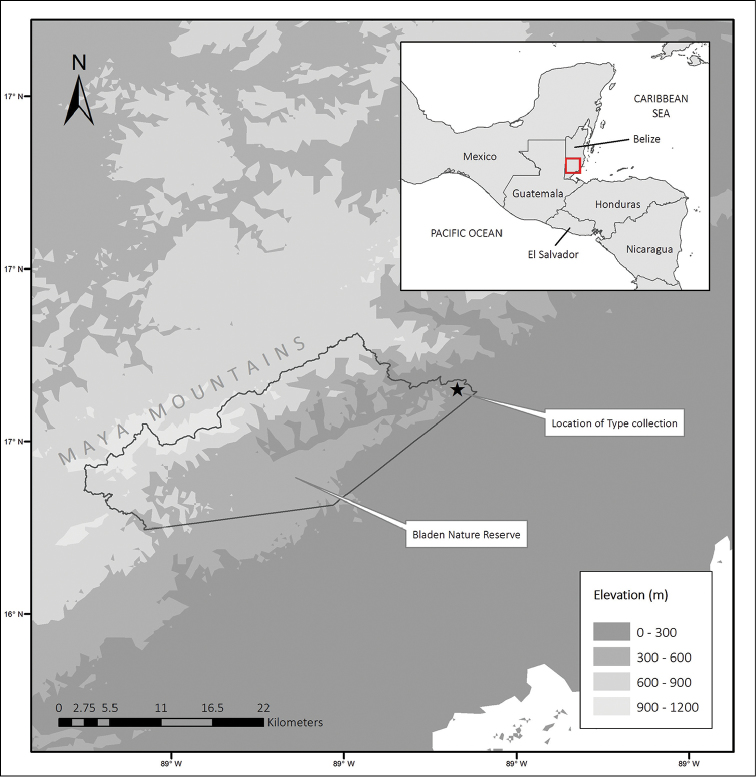
Elevation map of Belize with the type location (*) within the Bladen Nature Reserve.

Floristic inventories in the Bladen Nature Reserve, Belize, Central America (Figure 1) from 2012–2014 resulted in the discovery of this species of Lauraceae. Fertile herbarium material collected from the type location was determined to be unique among any of the known species of *Cinnamomum* by comparing it with type and other herbarium specimens housed at MO, and online at F (http://fm1.fieldmuseum.org/vrrc/), HUH (http://kiki.huh.harvard.edu/databases/specimen_index.html), NY (http://sweetgum.nybg.org/science/vh/), US (http://collections.nmnh.si.edu/search/botany/), and JSTOR Global Plants (https://plants.jstor.org/). This plant is described here as a new species, and a key to and notes on the *Cinnamomum* of Belize are provided. Images of the holotype and at least one isotype will be available on The Missouri Botanical Garden’s electronic database TROPICOS (http://www.tropicos.org). Our examination of recent material of the genus in Belize confirms the presence of *Cinnamomum areolatum* (Lundell) Kosterm. for the country, as first reported by Standley and Steyermark (1946, as *Phoebe areolata* Lundell).

## Taxonomic treatment

### Cinnamomum bladenense

Taxon classificationPlantaeLauralesLauraceae

S.W. Brewer & G.L. Stott sp. nov.

urn:lsid:ipni.org:names:60474708-2

#### Type.

BELIZE. Toledo District: Bladen Nature Reserve, c. 11 km north of Medina Bank, 16°33.14'N, 88°43.825'W, 320 m, 2 February 2016, *S. W. Brewer & G.L. Stott 7529*, (holotype, MO!; isotypes BM!, BRH!, CICY!, MO!, NY!, XAL)

#### Diagnosis.

*Cinnamomum bladenense* is morphologically similar to *Cinnamomum brenesii* (Standl.) Kosterm. from which it differs by its much smaller (c. 2.1 vs. 3 mm long) and campanulate (vs. urceolate) flowers, its inner tepals glabrous abaxially (vs. pubescent), its shorter petioles (< 10 vs. > 10 mm), its moderately and minutely sericeous (vs. tomentose) younger twigs, and its abaxially matte green (vs. light-green glaucous) mature leaves with clearly prominent secondary venation abaxially (vs. venation nearly plane with the lamina).

#### Description.

**Figure 2.**
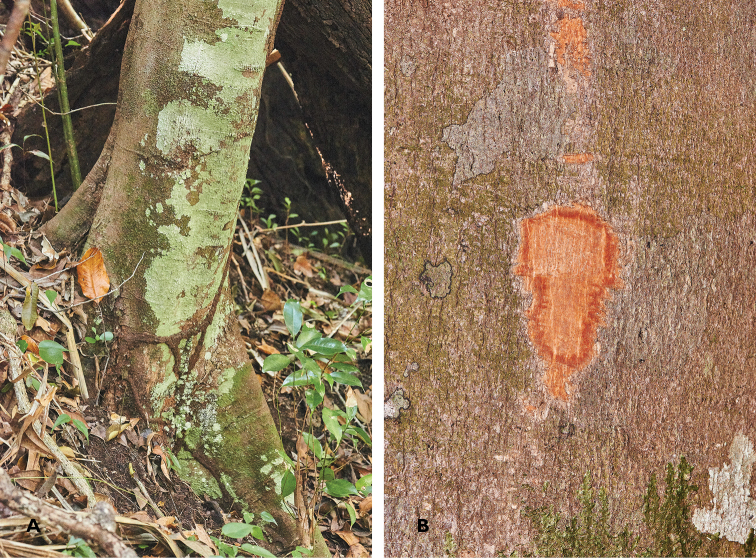
**A** Lower trunk and buttress of Cinnamomum bladenense **B** Bark and shallow trunk slash. Photos of same individual tree as *Brewer & Stott 7529* by SW Brewer 18 March 2014.

Tree 25 m tall, 26 cm DBH; bole round and mostly straight, with a low, narrow buttress of irregular-sized planks (Figure 2). Outer bark smooth, light-to-medium gray with a pinkish cast, occasional eye marks and rings, and lines of inconspicuous lenticels oriented lengthwise. Inner bark pinkish-brown with a moderately-pungent, chemical odor like that of bathroom cleaner (volatile, soapy).

Terminal buds moderately to densely sericeous with yellowish-white hairs. Twigs with slender, parallel, longitudinal grooves & low ridges (striate) to striate-angulate, less frequently laterally compressed and ridged-angulate, thinly to moderately-densely, minutely sericeous with silvery-white to yellowish, straight or weakly curved hairs 0.04–0.20 (0.3) mm long. Leaves alternate, thick-chartaceous to sub-coriaceous, ovate to ovate-elliptic, apex acute to shortly acuminate (rarely obtuse or rounded), base acute; within-branch leaf sizes highly variable, 11–31 mm wide × 35–86 mm long; petioles 3–9 mm, broadly and shallowly canaliculate, minutely sericeous. Venation mostly triplinerved, some subtriplinerved, the basal lateral nerves reaching c. ½ to ⅔ the length of the lamina; secondary veins 6–8; midvein and secondary veins immersed above, higher-order venation minutely impressed; midvein and secondary veins prominent below, higher order venation prominulous or minutely so. Inconspicuous domatia present in the form of barbellate axils of the basal-most pair of secondary veins, plane with the lamina, and usually present in one or two additional axils along the midrib, (rarely absent from a leaf). Laminae adaxially glossy, medium-dark green, glabrescent, with minute, mostly appressed to spreading, undulate to crisped, white to yellow-brown hairs on the basal portion of the midvein. Abaxial laminae light green, matte (to thinly and inconspicuously glaucous on young leaves), glabrescent or with very thinly-scattered, minute, subappressed and weakly undulate hairs; mid and lateral veins typically thinly minutely sericeous with appressed to subappressed hairs.

Inflorescences paniculate-cymose in leaf axils, 40–80 (130) mm, axes moderately covered in minute, mostly appressed (to spreading), straight to crisped hairs; bracts ligulate, mostly deciduous, to c. 5 mm long. Flowers narrowly campanulate, c. 2.0 mm long, on pedicels (2.0) 2.3–2.8 (-3.4) mm, drying dark brown to blackish-brown. Tepals 6, abscising nearly to the base in fruit, in flower spreading at shallow angles to the flower’s long axis, glabrous abaxially, sericeous adaxially, the margins basally ciliolate, ovate to broadly elliptic, outer c. 1.9 mm and inner c. 2.3 mm × c. 1 mm. Stamens 9, 0.8–1.4 mm, all four-celled, filaments pubescent on both surfaces, inner three with sub-globose glands at the base; staminodia 0.8–0.9 mm, filaments pubescent, heads cordate, to 0.5–0.6 × 0.3–0.4 mm. Pistil 1.1–1.9 mm, the style c. 10% longer than the ovary. Hypanthium sericeous inside, glabrous outside. Immature fruits thinly-glaucous green or green, ellipsoid to 10.3 × 6.0 mm. Cupules to 4.5 × 3.8 mm, tepal remnants inconspicuous, to 0.26 mm above the bottom of the sinus between tepal remnants, pedicels partly turbinate in fruit (Figure 4).

**Figure 3.**
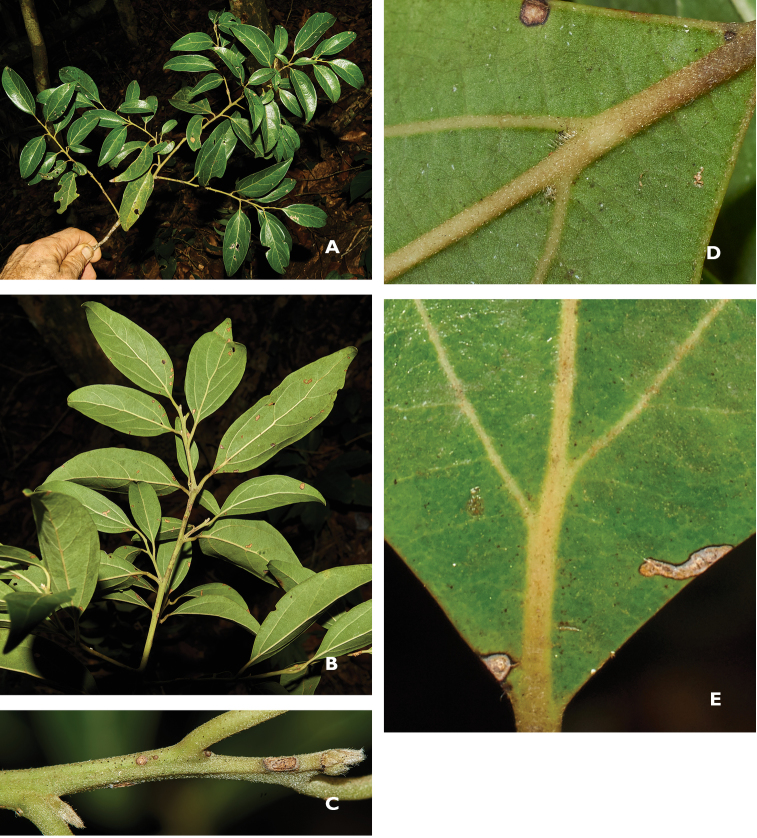
Diagnostic vegetative characters of Cinnamomum bladenense **A** Branch showing adaxial surface and variation of leaf sizes within branches **B** Abaxial surface of leaves showing matte green laminae and prominent midvein and secondary venation **C** Young twigs moderately and minutely sericeous with whitish buds **D, E** From the base of the same leaf lamina, respectively, adaxial surface of secondary vein axils not ampullous, abaxial surface of secondary veins barbellate and plane with the lamina surface. Photos from Brewer & Stott 7529 by SW Brewer 2 February 2016.

**Figure 4.**
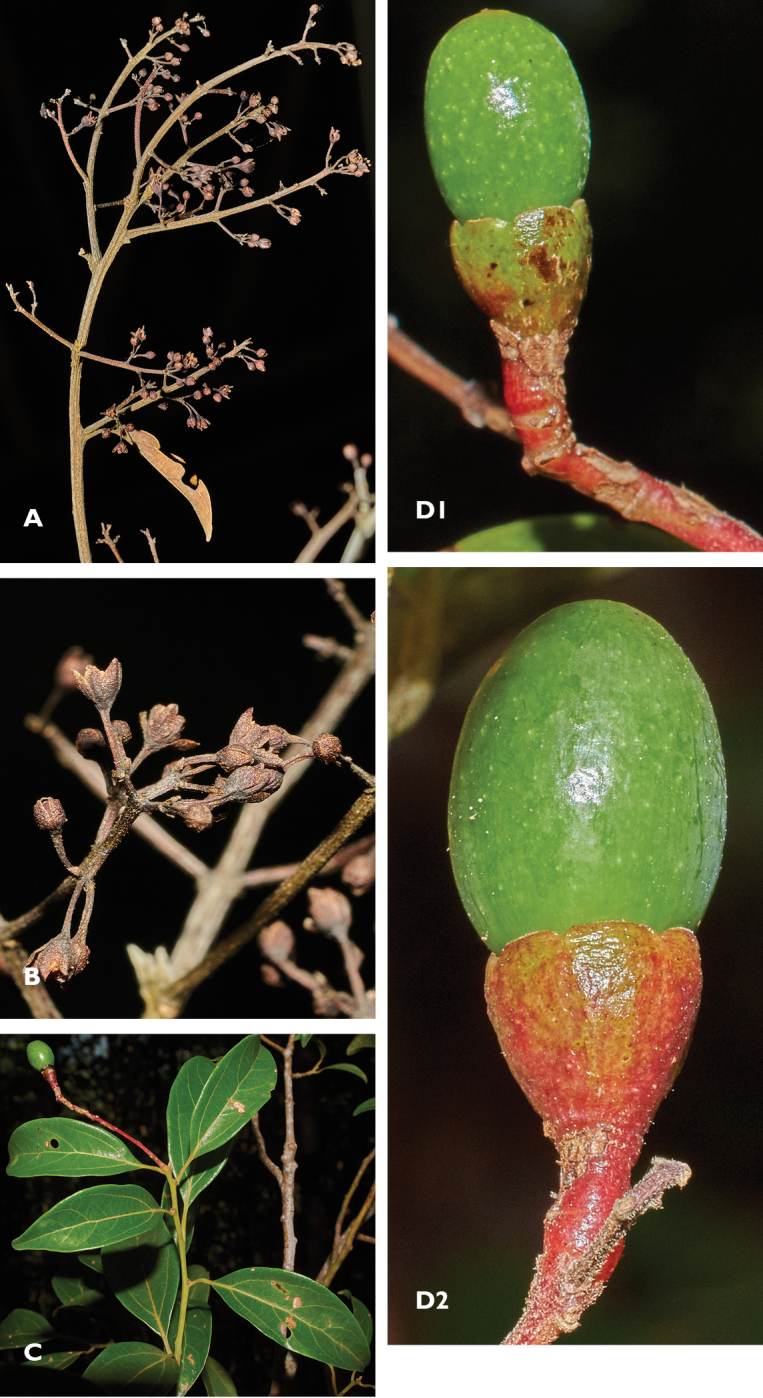
Diagnostic and other fertile characters of *Cinnamomum bladenense* **A** Axillary inflorescences on dry branch with all but one leaf removed to show variation in size and degree of branching **B** Apical portion of inflorescence showing flowers in bud and anthesis **C** Leafy branch with infructescence **D** Immature fruits with cupules showing partially turbinate pedicels, remnants of tepals at summit of the cupule, cupules turning red. Photos from *Brewer & Stott 7529* by SW Brewer 2 February 2016.

#### Other specimens examined.

*Brewer & Stott 7148* (from the same individual as the type, in sterile condition, collected in March 2014), *Brewer & Stott 6815* (sterile tree 15 m tall, 12 cm DBH, collected in December 2012).

#### Etymology.

The specific epithet honors the type location, the Bladen Nature Reserve, established in 1990 (IUCN Category 1a) to protect the watershed and the unique flora and fauna of the Bladen branch of the Monkey River. The origin of the word “Bladen” is unknown.

#### Phenology and reproduction.

Phenology data for this species are few; currently, flowering in this species is known to begin with the onset of the dry season, December-January, with fruits developing in January and February. The trees are not known to be fertile below 15 cm DBH and are not fertile every year.

#### Distribution and ecology.

*Cinnamomum bladenense* is currently known only from fewer than 10 individuals on two limestone ridge-and-knoll systems south of the Bladen branch of the Monkey River, a few km upstream from where the Bladen descends into the coastal plain. This canopy tree species occurs in semi-evergreen forest c. 25 m high on very-well-drained, steep and rocky slopes on Cretaceous limestone. Similar habitat occurs southwest of the type location nearly to the Guatemala border, and northeast of the type location to the southeastern portion of the Cockscomb Basin, along the southeastern foothills of the Maya Mountains.

#### Preliminary conservation assessment.

Population information is too limited to support an assessment of the extinction risk faced by *Cinnamomum bladenense*, and the category of Data Deficient (DD) is appropriate, according to IUCN (2012) criteria. The known habitat of the species is protected as part of a nature reserve, however anthropogenic fires and illegal logging in the area, including nearby potential habitat, are potential risks to the persistence of this species.

## Discussion

*Cinnamomum bladenense* is unique for the genus in its relatively small flowers (< 2.3 mm from the base of the hypanthium to the apex of the tepals of dried specimens), and for its occurrence in the canopy of relatively tall-canopied (20–35 m) limestone slope forest. It has not been found on the adjacent apices of well-exposed ridges or on exposed rock outcrops, which are comparatively lower- and more open-canopied, much denser, of proportionately smaller-trees, and more unique floristically (Brewer et al. 2003). Limestone forests in the type location and nearby forests of similar vegetation have a high proportion of limestone specialists (Brewer unpubl. data), with soil properties significantly different than nearby soils on volcanic substrata (Winbourne et al. 2016). As far as we know, this new species appears to be unique among Neotropical species, perhaps along with *C. salicifolium* (Nees) Kosterm. from Mexico, by being associated exclusively with limestone. The latter species has abaxially pubescent tepals persistent in fruit, conspicuous and often dense and/or often spreading indument on the leaves and young twigs, villous filaments abaxially, and occurs in oak-pine forests.

In Lorea-Hernández’ (1996) revision of the Neotropical species of *Cinnamomum* Schaeff., specimens of Brewer & Stott 7529 key to *C. brenesii*, to which it is most similar (see diagnosis for differences), to *C. paratriplinerve* Lorea- Hernández (ined.), and, with considerable latitude in interpretation of indument, *C. hartmannii* (I.M. Johnst.) Kosterm. *Cinnamomum brenesii* is a Costa Rican and Panamanian species most often collected in disturbed and open habitats such as pastureland, roadsides and trail edges (vs. closed-canopy limestone hill forests). *Cinnamomum paratriplinerve* differs from the new species in having external tepals glabrous adaxially, entirely turbinate pedicels (at least some) in fruit, hypanthium glabrous inside, and generally conspicuously longer leaves. It also is a species of Costa Rica and Panama in disturbed areas (pastureland, secondary forest) as well as “natural”, closed-canopy forest. *Cinnamomum hartmannii* is a species of northwestern Mexico, in the contact zone between dry, oak temperate forests and semi-deciduous to deciduous tropical forests. It has glabrous filaments, and mainly pinnate leaves villous-tomentose beneath when young, among other differences. All other known Neotropical species differ in one or more significant character states, including persistent or entirely-deciduous tepals, domatia absent or secondary domatia present, domatia of pocket-like depressions below and ampullous above, and indument of spreading to erect hairs.

The three species of *Cinnamomum* in Belize – *C. areolatum* (Lundell) Kosterm. *C. bladenense* S.W. Brewer & G.L. Stott, sp. nov., and *C. montanum* (Sw.) Bercht. & J. Presl – are easily separated in the field without fertile material. In Belize, *C. areolatum* is typically a small tree (usually < 10 m, < 10 cm DBH), treelet or colonial treelets/shrubs, on the tops and shoulders of ridges on very acidic and nutrient-poor, igneous substrate in somewhat open and low woody vegetation (woodlands or natural disturbances in low forest), dominated by or with a significant component of *Purdiaea belizensis* (A.C. Sm. & Standl.) J.L. Thomas, occasionally with *Cyrilla racemiflora* L. and sedges such as *Scleria* P.J. Bergius and *Rhynchospora* Vahl, and/or associated with areas of fire-dependent ferns [e.g., *Gleichenella pectinata* (Willd.) Ching, *Sticherus palmatus* (W. Schaffn. ex E. Fourn.) Copel., *Dicranopteris flexuosa* (Schrad.) Underw.)], and never on alluvium. Its canopy is somewhat scattered along the stem, not confined to the upper portion of the stem, with leaves that are < 5 cm wide, coriaceous to thick coriaceous, the upper surfaces smooth and plane, and the veins inconspicuously impressed above. The leaves have conspicuous, densely-prominulous reticulum beneath, and domatia that are pocket-like below and ampullous above (at least some leaves) in the axils of the basal (or more) secondary veins. *Cinnamomum montanum* is found in ± closed canopy forest on rich alluvium or on acid substrata, never limestone, and is quite capable of exceeding 10 cm DBH and 20 m in height. It has the largest leaves of the three species, very often ≥ 5 cm wide, with at least some leaves > 10 cm long (often some much longer). It also has conspicuous ampullous and pocket-like domatia; however, its upper surface is not plane but “quilted” (coarsely bullate above, with long concave areas beneath) from the impressed midvein and secondary veins. *Cinnamomum bladenense* is found only on limestone slopes, has small leaves with short petioles, inconspicuous domatia not ampullous, and is a tree with a tight canopy at the top of the stem, even when small.

### Key to Belizean *Cinnamomum*

**Table:** 

1	Tepals abaxially glabrous, deciduous in fruit. Flowers small, < 2.3 mm long from the base of the hypanthium to the apex of the tepals. Domatia plane with the leaf surface, inconspicuous. Trees on limestone soils, to 25 m and 25+ cm DBH	***C. bladenense***
–	Tepals abaxially pubescent or not, persistent in fruit. Flowers > 2.3 mm long. Domatia ampullous above (at least in some leaves), conspicuous. Trees on igneous substrata and/or alluvium.
2	Small trees, treelets, or low clumps of a few stems. Tepals abaxially glabrous in flower. Inflorescence axes and/or pedicels and flowers glaucous when young. Abaxial minor leaf venation dense and prominulous, leaves < 5 cm wide	***C. areolatum***
–	Trees, occasionally > 15 m and > 10 cm DBH. At least some tepals abaxially pubescent. Inflorescence axes and/or pedicels and flowers not glaucous. Abaxial minor leaf venation essentially flat, not dense, at least some (often most) leaves > 5 cm wide	***C. montanum***

## Supplementary Material

XML Treatment for Cinnamomum bladenense
